# COVID-19 and Pneumonia Five Years Later: New Evidence and Persistent Challenges

**DOI:** 10.3390/v17101298

**Published:** 2025-09-25

**Authors:** Zichen Ji, Javier de Miguel-Díez

**Affiliations:** 1 Respiratory Care Department, Infanta Leonor University Hospital, 28031 Madrid, Spain; zichen.ji@salud.madrid.org; 2 Respiratory Care Department, Gregorio Marañon General University Hospital, Gregorio Marañon Health Research Institute (IiSGM), 28007 Madrid, Spain; 3 Faculty of Medicine, Complutense University of Madrid, 28040 Madrid, Spain

The third edition of the Special Issue “COVID-19 and Pneumonia” includes nine original articles that provide relevant evidence on advances in the diagnosis, risk stratification, management, and follow-up of patients with coronavirus disease 2019 (COVID-19).

One article in this Special Issue compares the clinical, radiological, and evolutionary characteristics of patients with COVID-19 treated in 2020 and 2024 [1]. More recent cases exhibited a higher frequency of pneumonia and increased mortality, likely due to changes in population characteristics and diagnostic and treatment protocols. Similar variations have been reported by others, who have examined differences according to the predominant strain of the severe acute respiratory syndrome coronavirus 2 (SARS-CoV-2) in each period [2]. Due to this dynamic landscape, although COVID-19 has been known for over five years, scientific knowledge requires continuous updating to optimize clinical management.

The use of corticosteroid pulses in patients with COVID-19 has been extensively studied, although with inconsistent results across observational studies, clinical trials, and meta-analyses [3,4,5]. An observational study included in this Special Issue found that corticosteroid pulses did not improve the prognosis of critically ill patients and were instead associated with a higher incidence of secondary infections [6].

Also in the context of critically ill patients, another large observational study identified the main risk factors for severe disease progression and mortality [7]. Similarly, another study demonstrated that loss of muscle mass is an adverse prognostic factor [8], highlighting the importance of implementing nutritional and rehabilitation strategies in the management of these patients.

For predicting clinical outcomes, one study proposed combining the International Severe Acute Respiratory and Emerging Infectious Consortium (ISARIC) 4C mortality score with vaccination data and anti-S antibody titers. Low levels of these antibodies were associated with a greater risk of progression to severe COVID-19 [9]. Additionally, radiological predictive factors have been explored, such as the chest computed tomography severity score (CTSS), which is useful for estimating the risk of clinical deterioration [10]. These novel risk stratification tools allow for more precise assessment at hospital admission and complement other prognostic methods.

Beyond short-term prognosis, this Special Issue also includes studies addressing long-term outcomes. One study suggests that increased systolic pulmonary artery pressure in older and critically ill patients may represent a prognostic marker of long-term mortality [11]. In the post-infectious phase, lung ultrasound has been highlighted as an alternative to computed tomography for detecting residual pulmonary fibrosis, offering advantages such as greater accessibility and avoiding exposure to ionizing radiation [12].

In the laboratory setting, one article presents a Python tool named ct2vl, which enables the conversion of cycle threshold (Ct) values—widely used to date—into actual viral loads. This methodology facilitates data standardization and enhances comparability across studies and centers [13].

Altogether, the nine articles published in this Special Issue reflect the sustained effort of the scientific community to understand and address SARS-CoV-2 infection, even five years after the onset of the COVID-19 pandemic. Nevertheless, important challenges remain, including the heterogeneity of study populations, the need for external validation in multicenter studies, and the application of newly developed tools in resource-limited settings. Therefore, future research is required to overcome these limitations and consolidate current knowledge about COVID-19.
